# Three-Dimensional Perspective on Scar-Related Ventricular Tachycardia Substrate: Critical Conduction Abnormalities as Ablation Targets and Implications for Procedural Endpoints

**DOI:** 10.31083/RCM51668

**Published:** 2026-08-18

**Authors:** Yuki Komatsu, Akihiko Nogami

**Affiliations:** ^1^Department of Cardiology, Institute of Medicine, University of Tsukuba, 305-8577 Tsukuba, Japan; ^2^Department of Cardiology, Tokyo Heart Rhythm Hospital, 157-0063 Tokyo, Japan

**Keywords:** arrhythmia, ventricular tachycardia, electrophysiology, catheter ablation, endocardium, epicardium

## Abstract

Scar-related ventricular tachycardia (VT) is increasingly recognized as a three-dimensional (3D) arrhythmia sustained by complex reentrant circuits that frequently involve intramural components. Advances in high-density and high-resolution electroanatomical mapping have shifted the paradigm of substrate characterization from purely voltage-based structural definitions toward functional assessment of conduction delay and wavefront discontinuity. Recent studies have described techniques that target critical conduction abnormalities associated with VT circuits, including deceleration zones, lines of conduction block, and rotational activation patterns. A central concept underlying these approaches is that VT substrates are volumetric structures constrained by lateral and depth-oriented boundaries rather than planar pathways. Within this 3D framework, targeted ablation strategies that interrupt functionally relevant conduction abnormalities, rather than empirically modifying scar, have demonstrated clinical outcomes comparable to those of extensive homogenization approaches while potentially reducing procedural burden. Intramural reentry remains a major challenge because surface-based mapping incompletely captures mid-myocardial conduction, and current techniques for real-time intraprocedural assessment of intramural abnormalities remain limited. Adjunctive strategies, including differential pacing, refined annotation methods, and peak frequency analysis, may improve identification of critical substrate components, although no single parameter reliably defines the optimal ablation target. Accordingly, procedural endpoints should evolve beyond empirical surrogates such as VT termination or noninducibility toward confirmation of effective disruption of the 3D arrhythmogenic substrate. Continued integration of advanced mapping technologies, imaging, and lesion-delivery innovations will be essential to refine both ablation strategies and procedural endpoints in scar-related VT.

## 1. Introduction

Scar-related ventricular tachycardia (VT) remains a major cause of lethal arrhythmias in patients with both ischemic and nonischemic cardiomyopathies, and catheter ablation has become an essential therapeutic option for cases refractory to antiarrhythmic drug therapy [1,2,3,4]. Despite substantial advances in mapping and ablation technologies, VT recurrence remains common, underscoring persistent limitations in our understanding of the arrhythmogenic substrate and in the definition of procedural success. Successful ablation requires accurate elucidation of the mechanisms sustaining reentry and appropriate selection of an ablation strategy tailored to the underlying substrate. Traditionally, induction of VT, mapping during tachycardia, termination by ablation, and subsequent noninducibility have been regarded as hallmarks of procedural success. However, because many scar-related VTs are hemodynamically unstable, substrate modification performed during sinus rhythm has evolved as the central strategy in VT ablation [5,6,7,8,9,10,11].

Conventional substrate mapping has largely relied on a two-dimensional (2D) conceptual framework, focusing on the identification of low-voltage areas, late potentials, and fractionated electrograms. Despite these approaches, VT recurrence rates remain substantial, highlighting the need for a more fundamental understanding of the underlying reentrant circuit architecture. Advances in high-density and high-resolution mapping technologies have enabled more refined characterization of conduction delay, wavefront discontinuity, and critical isthmus regions during sinus or paced rhythm [12,13,14]. Furthermore, emerging evidence from high-density mapping and simultaneous endocardial–epicardial studies indicates that VT circuits frequently exhibit complex three-dimensional (3D) architectures involving subendocardial, intramural, and subepicardial components [15]. These observations challenge the traditional planar model of VT reentry and suggest that many apparent surface findings may represent projections of deeper volumetric circuits.

In parallel with this evolving understanding of 3D VT circuits, ablation strategies have progressively moved from empiric scar modification toward targeted approaches aimed at interrupting mechanistically defined critical conduction abnormalities. Nevertheless, important questions remain regarding how best to identify these abnormalities, how extensively ablation should be delivered, and how procedural success should be assessed when the arrhythmogenic substrate itself is 3D.

In this review, we integrate emerging insights into functional and 3D arrhythmogenic substrates with contemporary substrate-based ablation strategies and discuss how these concepts reshape both targeting strategies and procedural endpoints in scar-related VT ablation.

## 2. Classical Concepts of Scar-Related VT and Their Limitations

VT reentrant circuits have been conventionally conceptualized as 2D structures composed of a narrow isthmus bordered by two electrically unexcitable barriers. This schematic representation has long served as the dominant framework for understanding VT mechanisms and has been clinically supported by findings from entrainment mapping studies [16,17]. With the introduction of 3D electroanatomical mapping systems, early ablation strategies focused on visualizing activation patterns on the endocardial surface using color-coded maps during VT, thereby identifying ablation targets based on tachycardia activation sequences [18]—an approach commonly referred to as a “VT activation-guided strategy”. However, as VT is often hemodynamically intolerable, substrate-based ablation during sinus rhythm or paced rhythm has become the predominant contemporary strategy.

**Fig. 1.**
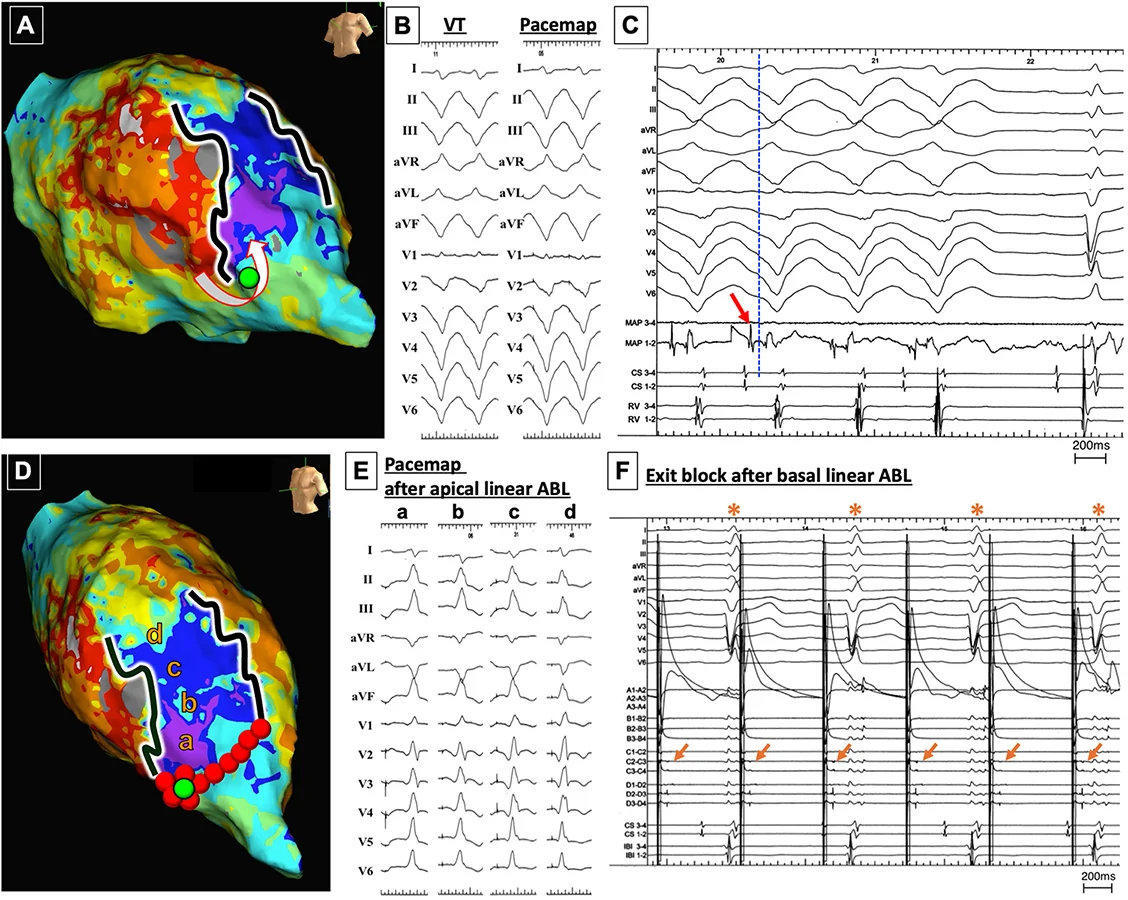
**Substrate–guided ablation and core isolation**. (A) Activation map during sinus rhythm demonstrated two lines of block (LOB) on the anterior wall. A rotational activation pattern (RAP) with markedly slow conduction was identified at the apical edge of the septal LOB. (B) Perfect pace mapping corresponding to the clinical VT was obtained at the site exhibiting the RAP (green tag). (C) During clinical VT, a presystolic potential (red arrow) was recorded at the same site, and radiofrequency ablation (ABL) resulted in immediate termination of the VT. (D) In this case, multiple VT morphologies were inducible. Because extensive local abnormal electrograms were observed within the low-voltage region of the anteroseptal wall, a core isolation strategy targeting the abnormal substrate was planned. Linear ablation was first performed to connect the apical edges of the two LOBs. (E) After completion of the apical edge-to-edge linear lesion, pace mapping was performed at sites a–d. At site a, which had initially demonstrated a perfect pace map with a superior axis morphology, the QRS axis shifted to an inferior axis. Moreover, QRS morphologies obtained at sites b, c, and d were nearly identical, and the S–QRS interval was shorter at the basal sites, suggesting preferential exit of activation through the basal region between the two block lines. (F) After a second linear lesion was created connecting the basal edges of the two LOBs, pacing from the catheter positioned within the isolated region demonstrated local capture without conduction outside the region (orange arrow and asterisk), confirming exit block and successful completion of core isolation. The two LOBs represented fixed conduction barriers. All VTs became noninducible after core isolation. VT, ventricular tachycardia.

Histopathological investigations of explanted human hearts have provided important insights into the structural basis of post-infarction VT. Surviving myocardial fibers may form interconnecting channels within the borders of fibrotic tissue and can function as slow-conducting pathways that support reentrant VT circuits [19,20]. Based on this understanding, surgical subendocardial resection targeting abnormal and fractionated electrograms demonstrated favorable long-term arrhythmia-free outcomes, with reported success rates of approximately 60–80% in patients with prior myocardial infarction [21]. These surgical experiences laid the conceptual foundation for catheter-based substrate ablation strategies, in which abnormal electrograms within scar tissue are identified and ablated [5,6,7,8,9,10,11]. Electrically isolating the low-voltage scar segment that constitutes the core of the ventricular tachycardia substrate–with confirmation of exit block–represents an effective strategy rather than ablating the entire scar (Fig. 1).

Bipolar voltage mapping has been widely adopted to delineate dense scar and the scar border zone. Nevertheless, there are several important limitations of voltage-based scar definition. Bipolar voltage amplitude is highly dependent on electrode size, interelectrode spacing, and the direction of wavefront propagation, and it lacks the specificity required to distinguish arrhythmogenic scar from non-arrhythmogenic tissue [22,23]. Consequently, the simplistic assumption that low-voltage regions directly correspond to critical arrhythmogenic substrate has proven inadequate, contributing to the adoption of extensive scar homogenization approaches.

Late potentials and local abnormal ventricular activities (LAVA) depend on the recording site and the direction and sequence of activation during mapping [23,24]. These electrogram features may arise not only from components of the VT circuit but also from collision of activation wavefronts propagating from different directions, even at sites unrelated to the reentrant pathway [25]. As a result, reliance on late potentials alone has been shown to have limited specificity for identifying critical components of VT circuits. In addition, extensive scar homogenization represents a “probabilistically robust” strategy, in that widespread lesion delivery increases the likelihood of intersecting at least one critical component of a 3D reentrant circuit [10]. However, this approach entails significant procedural and clinical burdens, including increased lesion burden, prolonged procedural duration, potential adverse effects on ventricular function, and a potentially higher risk of complications.

Importantly, these limitations stem from a fundamental conceptual issue: voltage-based approaches primarily describe structural anatomy rather than the dynamic properties of conduction. Bipolar voltage amplitude reflects the presence of scar but does not directly characterize the spatial distribution of conduction delay, wavefront discontinuity, or functional barriers that sustain reentry. As a result, anatomical scar definition alone may incompletely identify the critical components of a VT circuit.

These considerations have prompted a paradigm shift from purely structural substrate mapping toward functional characterization of conduction dynamics, enabled by high-density and high-resolution mapping technologies.

## 3. High-Density/High-Resolution Mapping and Functional Substrate

Advances in high-density and high-resolution mapping technologies have provided a new perspective for defining arrhythmogenic substrates based on functional properties, particularly conduction delay and conduction discontinuity [12,13,14]. This paradigm shift—from purely structural substrate mapping toward functional substrate mapping—has drawn increasing attention as a strategy to identify myocardial regions more specifically associated with VT circuits. By focusing on electrophysiological characteristics rather than voltage amplitude alone, functional mapping aims to localize substrates that are more likely to represent critical components of reentrant tachycardia.

Irie, Tung, and colleagues [26] introduced Isochronal Late Activation Mapping (ILAM), a technique in which ventricular activation during sinus rhythm is temporally divided into eight equal isochronal segments to visualize localized conduction slowing as isochronal crowding. Using this approach, they demonstrated that regions of delayed activation can be clearly identified during sinus rhythm and that these regions may represent arrhythmogenic substrates independent of bipolar voltage amplitude [26]. Areas in which three or more isochrones were crowded within a distance of 1 cm were defined as deceleration zones (DZs) and were proposed as critical substrates identifiable during sinus rhythm [13]. DZs were present at 95% of sites where VT termination was achieved, and reproducibility of DZ location was observed in 85% of cases even when mapping was repeated using different pacing sites. Ablation targeting DZs resulted in freedom from VT recurrence in approximately 70% of patients during a mean follow-up of 12 ± 10 months [13].

**Fig. 2.**
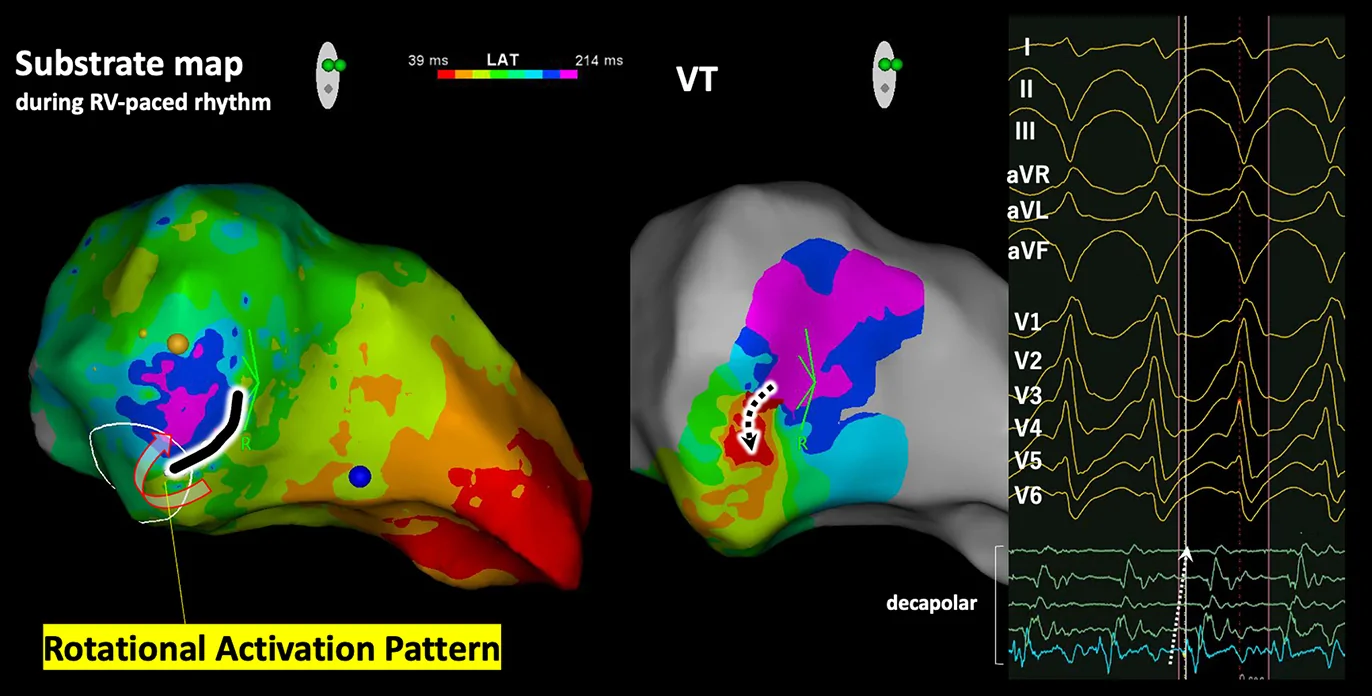
**Rotational activation pattern at the VT critical isthmus**. During substrate mapping under right ventricular (RV) pacing, a rotational activation pattern (RAP) was identified pivoting around the edge of a conduction block at the basal interventricular septum of the left ventricle (left panel). The critical isthmus of the VT colocalized with this site, where a diastolic potential was recorded during VT (middle and right panels). Radiofrequency ablation at this location resulted in termination of the VT. LAT, local activation time.

**Fig. 3.**
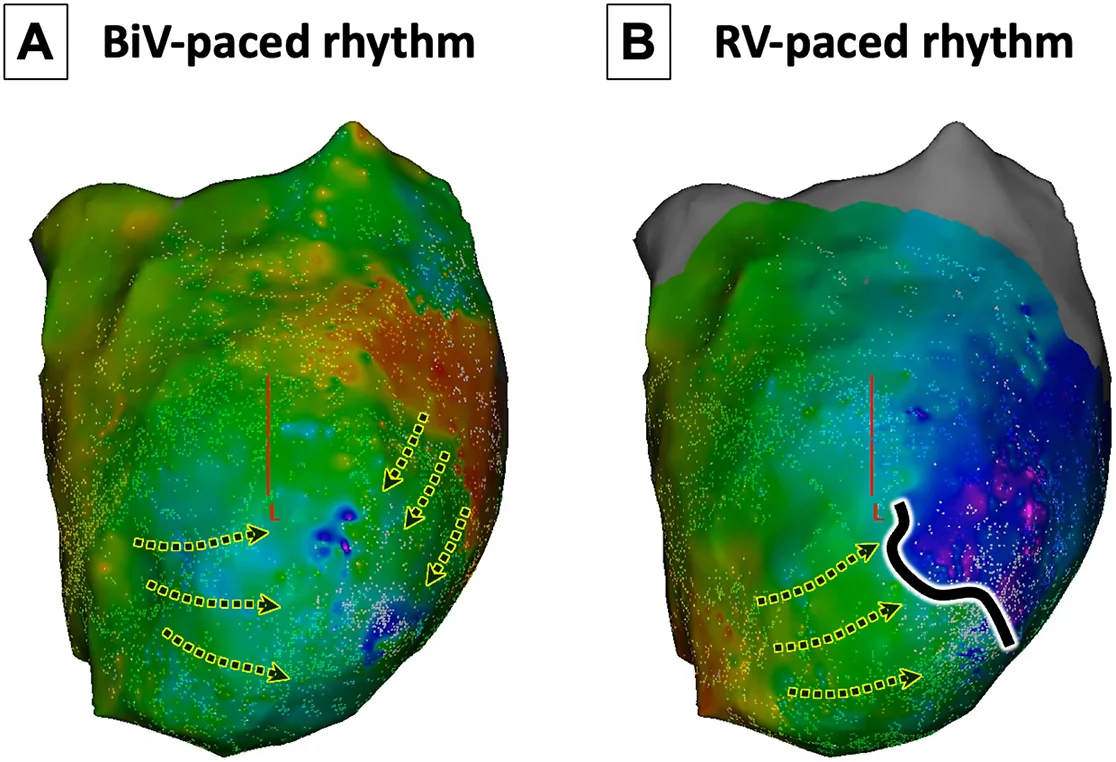
**Wavefront direction and manifestation of a line of conduction block**. (A) During biventricular-paced rhythm, activation wavefronts originating from the septal and lateral walls collided at the anterior wall, obscuring the line of conduction block. (B) During right ventricular–paced rhythm, the line of conduction block became clearly apparent. BiV, biventricular.

In our experience with functional substrate mapping, sites associated with VT termination frequently demonstrated a rotational activation pattern (RAP), pivoting around the edge of a conduction block during baseline sinus or ventricular-paced rhythm (Fig. 2). When the relationship between RAP and VT circuits was systematically evaluated, RAP was identified in approximately 70% of 33 VT morphologies in which VT termination by ablation or a perfect pace map with subsequent noninducibility was achieved [14]. A key feature of RAP is that it reflects not merely conduction delay but a rotational propagation pattern characterized by inward curvature of the wavefront pivoting around the edge of a conduction block. These electrophysiological characteristics suggest the presence of a localized line of conduction block forming the boundaries of the reentrant circuit. Among patients in which RAP was not apparent during mapping in sinus rhythm, additional mapping performed under ventricular pacing revealed newly emergent RAP in a subset of patients [27]. Mapping during pacing near the region of interest facilitates identification of the RAP and the line of conduction block, as delineation becomes difficult when activation wavefronts approaching from multiple directions converge and collide within the region (Fig. 3).

As a functional substrate mapping approach that captures dynamic electrophysiological properties, one strategy involves identifying sites exhibiting decrement-evoked potentials during ventricular extrastimulation [28,29]. Furthermore, dynamic substrate mapping during extrastimulation may allow more specific identification of arrhythmogenic substrate than conventional substrate mapping [30,31,32,33].

**Fig. 4.**
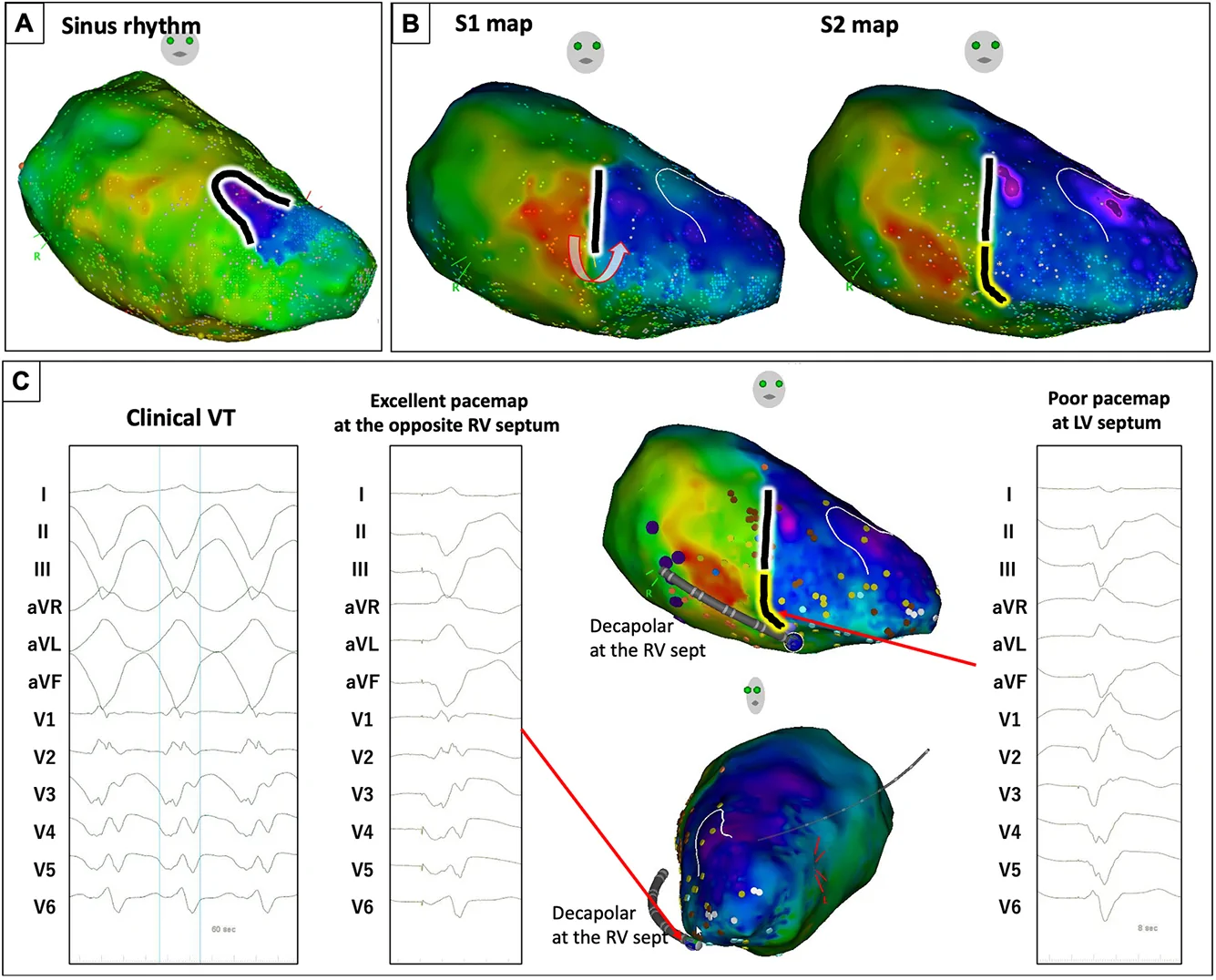
**Line of conduction block and identification of the primary VT target**. (A) During sinus rhythm, a U-shaped line of block (LOB) was identified at the apical anterior wall. (B) In contrast, during right ventricular (RV)–paced rhythm, a LOB was observed at the mid-septal region (S1 map). During extrastimulation from the same pacing site (S2 map), a decrement-evoked potential was recorded at the site that had exhibited a rotational activation pattern (RAP) at the edge of the LOB on the S1 map, resulting in extension of the LOB. (C) The clinical VT demonstrated a poor pace map at the RAP site identified on the S1 map but showed an excellent pace map at the opposite RV septum. The U-shaped LOB observed during sinus rhythm was not related to the clinical VT, whereas the LOB identified on the S1 map represented the primary target associated with the clinical VT. LV, left ventricular.

When conduction abnormalities such as isochronal crowding, DZ, lines of conduction block (LOB), or RAP are identified during substrate mapping, it is essential to perform pace mapping to determine which sites represent critical conduction abnormalities related to the targeted VT. Because multiple regions exhibiting conduction abnormalities may coexist within the substrate, careful determination of the primary target associated with the targeted VT is of paramount importance (Fig. 4). During pace mapping, particular attention should be paid to sites where the QRS morphology abruptly changes from a poor to an excellent match [34,35], as well as to locations demonstrating multiple exit sites (MES), characterized by the emergence of more than one QRS morphology during pacing from the same site [36].

## 4. Three-Dimensional Arrhythmogenic Substrates

VT circuits confined to a purely 2D plane are relatively uncommon. Simultaneous endocardial and epicardial mapping (SEEM), introduced by Tung and colleagues, represents a landmark investigation that directly demonstrated the 3D nature of VT circuits in humans [15]. Using SEEM, the majority of VT circuits involve nonuniform transmural conduction, rendering it difficult to fully delineate the reentrant pathway using surface-based mapping alone. A substantial proportion of VTs are sustained by entirely intramural (mid-myocardial) reentry, in which focal activation patterns are observed on both the endocardial and epicardial surfaces.

Building upon these observations, Nishimura and Tung proposed a conceptual framework in which VT circuits are understood as 3D hyperboloid structures rather than planar pathways [37]. In the hyperboloid model, the VT isthmus is defined as a 3D structure with hyperbolic geometry, and the reentrant circuit is constrained by fixed LOBs that form both lateral and depth boundaries. From this perspective, LOBs identified during sinus rhythm should not be interpreted merely as 2D barriers on a mapping surface, but rather as manifestations of depth-oriented boundaries within a 3D VT circuit. The presence of concealed LOBs that become apparent only with differential pacing maneuvers [37]. This concept is in line with the observation that RAP may be unmasked depending on the direction of the activation wavefront relative to the scar during mapping [27]. Therefore, substrate mapping findings such as LOBs, RAP, and DZs should be interpreted not as isolated surface phenomena, but as surface projections of a volumetric 3D circuit.

## 5. Ablation Strategies Based on Three-Dimensional Arrhythmogenic Substrates

If VT circuits are understood as volumetric 3D structures rather than planar pathways, ablation strategies must be aligned with this conceptual framework. The concept of 3D VT circuits represents more than a theoretical advance; it carries important clinical implications that fundamentally reshape substrate-based ablation strategies. In particular, this framework provides a mechanistic explanation for why surface-limited ablation alone is often associated with suboptimal VT-free survival and why high-power or deep lesion formation may be required in selected cases. When the hyperboloid model of VT circuits is considered, identification of a LOB during sinus rhythm or paced rhythm suggests that ablation targeting regions adjacent to both sides of the LOB may be advantageous. We suggest that in patients in whom RAP around the LOB associated with a targeted VT is identified, localized ablation encompassing the RAP region may represent a rational ablation strategy [38]. Identification and intensive ablation of targeted RAP areas that were identified with high-density and high-resolution substrate mapping resulted in a high probability of freedom from VT recurrence, exceeding 80% in reported series [38].

When an intramural isthmus contributes to VT maintenance, conventional radiofrequency energy delivery from the endocardial or epicardial surface may be insufficient to create a transmural lesion. In such cases, it is crucial to identify segments of the critical isthmus that course closer to the mapped chamber surface, rather than targeting activation gaps located deep within the myocardial wall. These surface-adjacent components may represent more accessible and effective ablation targets within an otherwise intramural circuit.

**Fig. 5.**
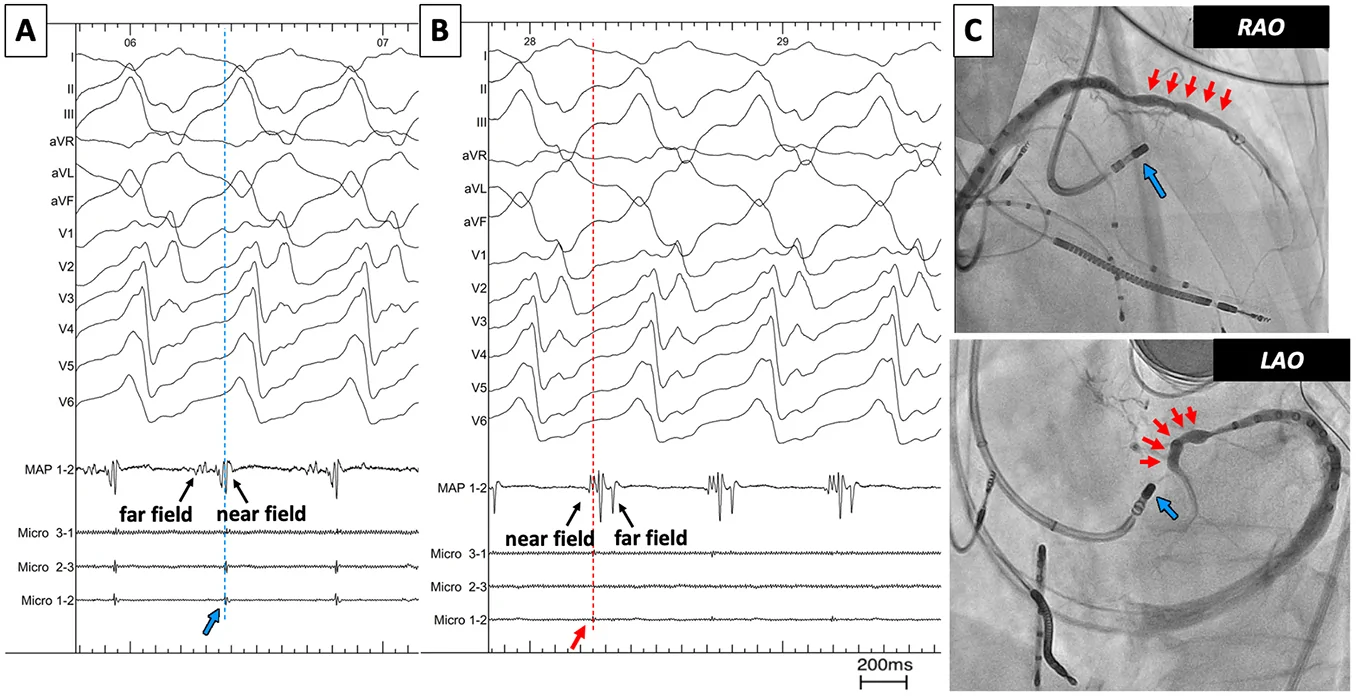
**Microelectrode recordings reflect true near-field potentials**. (A) The conventional bipolar electrogram recorded by an ablation catheter (QDOT Micro, Biosense Webster) exhibited multiple components. The microelectrode potentials occurred at the timing of QRS onset (blue arrow). Among the multiple components observed on the conventional bipolar electrogram, the component coinciding with QRS onset was considered the near-field potential, whereas the component preceding the QRS was interpreted as a far-field potential. (B) When the ablation catheter was advanced to the contralateral coronary venous system (red arrow), the timing of the near-field potential shifted, corresponding to a presystolic potential (red arrow), consistent with local activation at that site. (C) Fluoroscopic view demonstrating the catheter position during panel A (blue arrow). The catheter was positioned within the contralateral coronary venous system (red arrow) during (B). RAO, right anterior oblique; LAO, left anterior oblique.

To effectively interrupt conduction of the VT circuit, particular attention should be paid to the near-field components of bipolar electrograms. When using the QDOT Micro catheter (Biosense Webster) under the guidance of the CARTO3 system (Biosense Webster), the microelectrodes are designed to minimize far-field contamination and thus more accurately reflect true local activations. Consequently, the recorded signals predominantly represent activation of the immediately adjacent myocardial surface being mapped, and multiple-component or fractionated potentials are less frequently observed (Fig. 5).

**Fig. 6.**
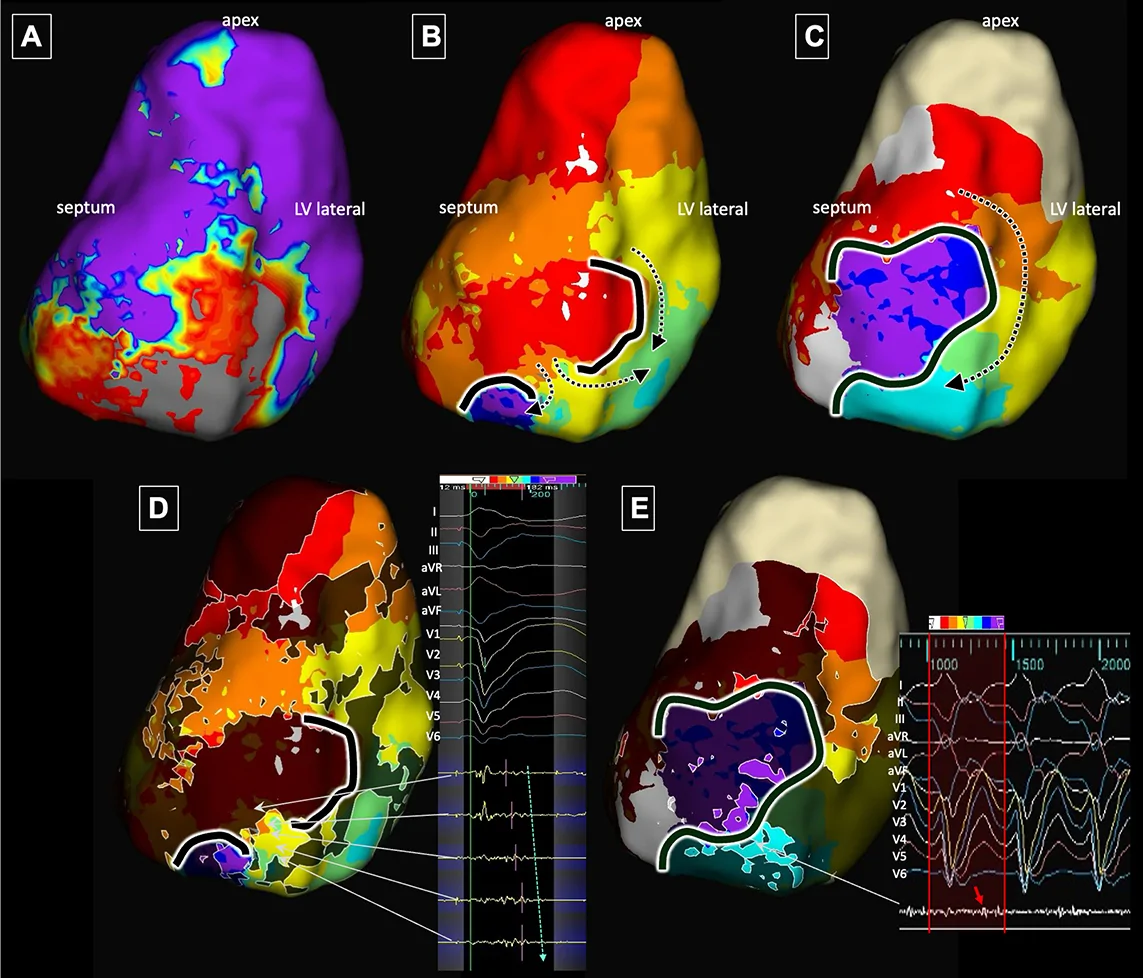
**Peak frequency–guided identification of the ablation target of a three-dimensional VT circuit**. (A) Voltage mapping demonstrated a low-voltage area at the basal inferior wall. (B) During right ventricular (RV)–paced rhythm, a line of block (LOB) was identified within the basal LV low-voltage area. A slow-conduction gap traversing the region between two LOBs was observed immediately proximal to the latest activation site. (C) During VT, activation propagated from the apical region along the lateral LV toward the basal region following the LOB. No further conduction was observed beyond the light blue timing, suggesting that activation from the basal LV descended intramurally and re-emerged apically, consistent with a three-dimensional VT circuit. (D,E) When regions with a peak frequency ≥300 Hz were highlighted, the areas identified during RV-paced rhythm and during VT were concordant within the low-voltage region. A mid-diastolic potential was recorded at this site during VT, and radiofrequency ablation resulted in immediate termination of the VT.

Using the EnSite mapping system (Abbott), regions exhibiting markedly high peak frequency along the VT circuit may reflect dominant near-field components recorded at the myocardial surface and may correspond to locally vulnerable areas. In contrast, segments of the circuit that are predominantly intramural may manifest as lower peak frequency values in recorded local ventricular potentials. Channels in which surviving superficial myocardium is strongly involved—and in which ablation lesions are more readily delivered—exhibit higher peak frequency values (Fig. 6), with thresholds above approximately 405 Hz associated with rapid VT termination during radiofrequency application [39]. Sites corresponding to the VT isthmus have been shown to exhibit both low bipolar voltage and elevated peak frequency during sinus rhythm or paced rhythm [40]. However, comparative analyses of electrograms from dominant VT circuits and bystander scar regions have demonstrated no significant difference in peak frequency between isthmus and nonparticipating scar tissue, indicating that peak frequency alone cannot reliably discriminate critical VT isthmuses from bystander substrates [41]. Accordingly, peak frequency should not be regarded as a standalone determinant of ablation targets but rather as an adjunctive parameter that must be integrated with circuit-dependent information, such as activation or entrainment mapping, or other functional substrate markers.

## 6. Approaches to Assess Intramural Arrhythmogenic Substrate

Despite advances in high-resolution surface mapping, direct assessment of intramural conduction abnormalities remains one of the most significant unresolved challenges in VT ablation. When a component of a 3D VT circuit is located along the endocardial or epicardial surface, mapping may allow identification of a LOB corresponding to the depth boundary of the reentrant pathway. As discussed above, accurate delineation and ablation of LOB or regions of isochronal crowding during sinus rhythm can therefore be effective in interrupting VT circuits. In contrast, mid-myocardial reentry—characterized by absence of isthmus activation on both the endocardial and epicardial surfaces—often manifests as focal activation patterns on both surfaces. In such cases, LOB is frequently indistinct, rendering circuit interruption by conventional ablation strategies particularly challenging [38].

When substrate mapping fails to reveal clear wavefront discontinuity and a predominantly intramural mid-myocardial arrhythmogenic substrate is suspected, the following practical considerations may guide clinical decision-making.

### 6.1 Attention to Activation Gaps during VT Activation Mapping

During VT activation mapping, sites demonstrating an activation gap within the VT cycle length—particularly around lines of conduction block, when identifiable—may suggest intramural conduction. This is especially relevant when the activation gap corresponds to the diastolic phase, indicating that the critical isthmus may be located intramurally. Based on these findings, the likely VT circuit can be conceptualized, and ablation targets can be selected accordingly.

### 6.2 Attention to Far-Field Potentials

**Fig. 7.**
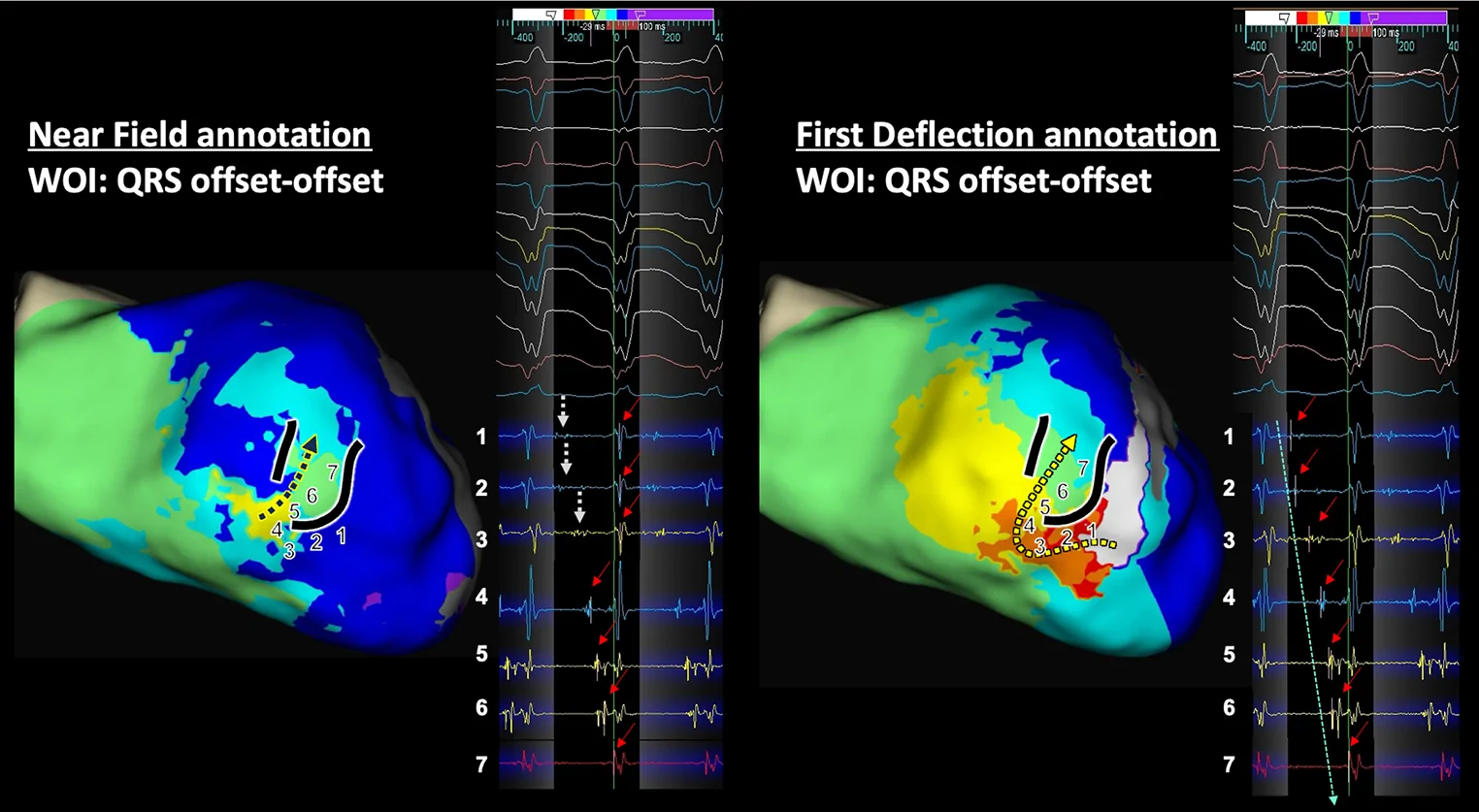
**Annotation of the diastolic potentials during VT activation mapping**. Near-field annotation failed to annotate the non-sharp mid-diastolic potentials recorded at sites 1–3 (white dotted lines), which were presumed to represent far-field potentials (left panel). In contrast, when first-deflection annotation was applied, the activation sequence could be tracked from sites 1 through 7, and the map clearly demonstrated diastolic-phase activation propagating around the line of block (right panel). WOI, window of interest.

One approach to inferring the presence of intramural substrates involves careful assessment of far-field ventricular potentials. Ideally, accurate mapping requires precise delineation of local activation within scar tissue, which is best achieved by minimizing far-field effects and recording near-field ventricular electrograms using small electrodes with short interelectrode spacing [42,43,44]. However, several prior case reports have suggested that far-field ventricular signals may provide valuable information regarding VT activation occurring deeper than the mapped surface [45,46]. Such intramural activity may be preferentially accentuated in bipolar electrograms recorded with relatively wider interelectrode spacing. During VT mapping, adjustment of the window of interest to the diastolic phase, or annotation of the first deflection relative to the offset of the surface QRS complex, may allow capture of pre-systolic or diastolic far-field components originating from intramural activation (Fig. 7).

**Fig. 8.**
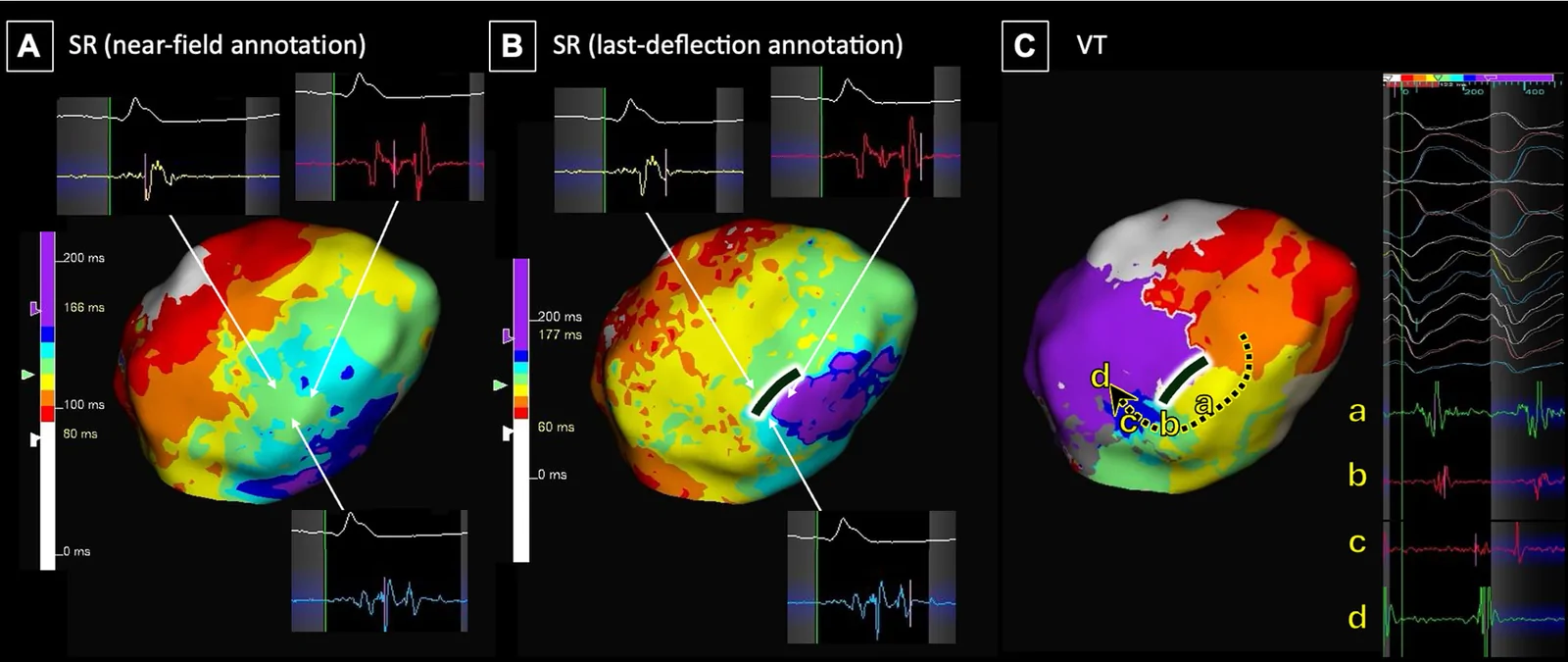
**Visualization of the deceleration zone by changing the annotation strategy**. (A) During sinus rhythm (SR), near-field annotation failed to clearly delineate the deceleration zone. (B) When annotation was performed at the last deflection, including far-field components of the local electrograms, the deceleration zone became clearly apparent. (C) VT activation propagated through an isthmus corresponding to the deceleration zone identified by last-deflection annotation during SR.

In substrate mapping, a similar concept can be applied. Although conventional 3D mapping strategies typically aim to minimize far-field effects in favor of near-field signal annotation, attention to abnormal far-field components—particularly delayed components or signals recorded in the temporal interval between split potentials—may provide complementary insights into intramural conduction abnormalities. Using the EnSite mapping system (Abbott), annotation of the offset of local ventricular electrograms can facilitate visualization of sites exhibiting intramural conduction abnormalities (Fig. 8). Isochronal crowding or LOB observed on surface maps should not necessarily be interpreted as representations of surface activation, but rather as projections of intramural conduction patterns onto the mapped chamber surface.

### 6.3 Mid-Myocardial Mapping Using a Microcatheter

Direct mapping of intramural components of VT circuits has also been attempted using specialized techniques, such as insertion of guidewires or microelectrodes into small branches of the coronary venous system [47,48]. In particular, mapping of the interventricular septum using a microcatheter advanced into septal perforator branches via the distal coronary sinus may facilitate direct assessment of intramural activity. Furthermore, the interventricular septum can be mapped using over-the-wire catheters, such as the EPstar Fix AIV (2.7 Fr; Japan Lifeline, Tokyo, Japan), not only via coronary venous branches but also through selected branches of the coronary arterial system [49]. Nevertheless, real-time, comprehensive visualization of intramural conduction dynamics remains beyond the full capability of current clinical mapping systems.

### 6.4 Preprocedural Cardiac Imaging

Preprocedural cardiac magnetic resonance imaging can be useful to evaluate the distribution of mid-myocardial scar and to support the identification of intramural substrate. Late gadolinium enhancement cardiac magnetic resonance imaging (LGE-MRI) represents the most robust modality for assessing the presence and spatial distribution of intramural scar, enabling preprocedural identification of 3D substrates that are not fully captured by surface-based mapping alone [50,51,52]. In particular, LGE-MRI provides unique insights into intramural scar architecture, which plays a central role in sustaining complex VT circuits. Preprocedural MDCT provides complementary anatomical and structural information, including wall thickness and scar distribution, and aids in identifying critical structures to support procedural planning and safety [53,54].

## 7. Rethinking Procedural Endpoints

The concept of procedural endpoints in VT ablation warrants reconsideration in light of emerging insights into three-dimensional arrhythmogenic substrates.

Although VT noninducibility remains a widely used procedural endpoint, it has important limitations and is insufficient as a standalone marker of success. Within a three-dimensional framework, endpoints should focus on effective disruption of critical conduction abnormalities, supported by post-ablation remapping. However, in predominantly intramural substrates, where surface mapping is limited, VT termination and noninducibility may remain the only practical indicators of success. In this section, we discuss the conceptual framework of procedural endpoints in VT ablation.

### 7.1 The Role of VT Noninducibility

Noninducibility of VT assessed by programmed electrical stimulation (PES) represents the procedural endpoint with a long history and substantial supporting evidence [55,56,57,58]. From a clinical standpoint, VT noninducibility offers a practical and readily assessable marker of acute procedural success and therefore continues to be widely adopted as a procedural endpoint. Nevertheless, reliance on VT inducibility assessed by PES is subject to several important limitations. VT induction is influenced by the effects of antiarrhythmic medications, alterations in autonomic tone related to sedation or general anesthesia, and variability in stimulation sites and pacing protocols. Moreover, repeated PES performed across multiple longitudinal evaluations may increase the likelihood of VT induction [59]. Conversely, substrate mapping—an indispensable component of current ablation strategies for scar-mediated VT—itself may modify VT inducibility through mechanical and electrophysiological effects on the myocardium [60].

In addition, because most patients with scar-related VT have concomitant heart failure and develop hemodynamic instability during VT, post-ablation PES carries a non-negligible risk of inducing nonclinical arrhythmias, particularly rapid monomorphic VT or polymorphic VT, which may adversely affect hemodynamic status. This concern is especially relevant in patients with high-risk profiles as defined by the PAINESD risk score (Pulmonary disease, Age, Ischemic cardiomyopathy, New York Heart Association (NYHA) functional class, Ejection fraction, Storm, Diabetes mellitus) [61] or the PAINES2D score, which incorporates scar volume as an additional parameter [62]. In such high-risk patients, aggressive pursuit of VT noninducibility through repeated PES should be avoided.

Furthermore, PES frequently induces nonclinical VTs that have not been observed in the patient’s spontaneous clinical course [63]. Whether all inducible VTs—including those with relatively short cycle lengths—should be targeted for ablation, or whether ablation should be confined to regions associated with the clinical VT, remains a matter of debate. This decision may need to be individualized, taking into account overall patient frailty, procedural duration, and intraprocedural hemodynamic tolerance. Accordingly, although VT noninducibility remains an important and valuable procedural endpoint, it is insufficient as a standalone determinant of procedural success. Particularly in the context of substrate-based ablation as the primary strategy, there is a clear need to reconsider and supplement procedural endpoints beyond VT noninducibility alone.

### 7.2 Rethinking Ablation Endpoints Based on Three-Dimensional Substrate Characterization

Within a 3D conceptual framework, procedural success should no longer be defined solely by arrhythmia suppression, but by evidence of effective disruption of the critical conduction architecture sustaining the VT circuit.

Across multiple studies, complete elimination of abnormal or late potentials, as well as successful core isolation, has not been achievable in all patients; however, when such endpoints are attained, they are consistently associated with favorable VT-free survival [8,9,64,65,66,67,68,69,70,71,72,73,74]. Loss of myocardial capture during high-output pacing has been used as an indicator of sufficient lesion formation [10,65,75]. Although the optimal pacing output and pulse width required to define capture loss remain debated [76], the absence of capture with high-output pacing reasonably suggests local electrical unexcitability and provides supportive evidence of adequate lesion formation.

Clinical outcomes following intensive ablation of targeted regions encompassing DZ, wavefront discontinuity lines, and RAPs have demonstrated one-year freedom from VT recurrence rates of approximately 70–80% [13,38,77]. In patients with ischemic cardiomyopathy, VT-free survival rates approaching 80% have been reported using such targeted strategies, which are comparable to the 84% freedom from VT observed in ischemic cardiomyopathy patients undergoing extensive scar homogenization—despite the substantially longer ablation times required for homogenization [10].

These observations support the concept that effective modification of functionally defined conduction abnormalities—rather than indiscriminate scar elimination—may be sufficient to interrupt a volumetric reentrant circuit.

**Fig. 9.**
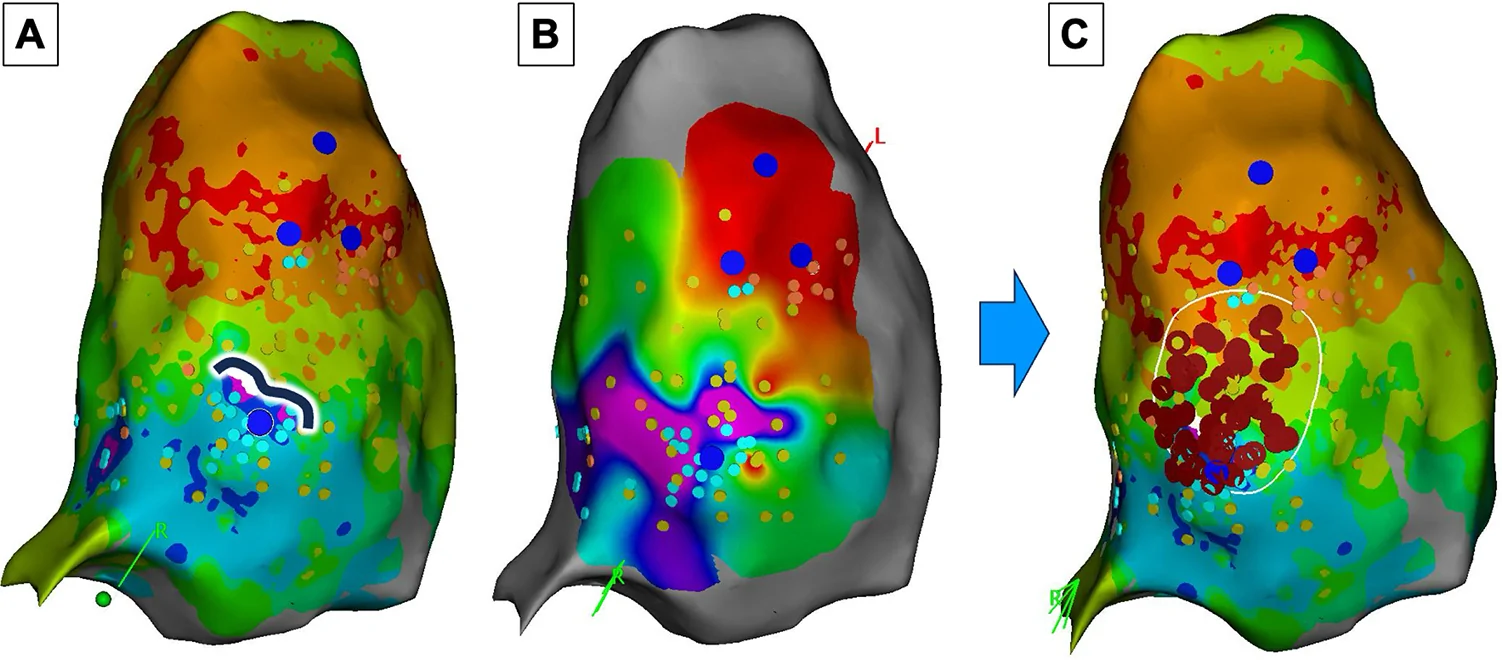
**Ablation of the area encompassing the critical conduction abnormality related to the targeted VT**. (A) Substrate mapping demonstrated a short line of block (LOB). (B) When the pace mapping score for the targeted VT was displayed on the map, an abrupt transition from a poor to an excellent match was observed along the LOB. (C) This site was considered a critical conduction abnormality related to the targeted VT. Ablation was performed at this location, including a surrounding area exhibiting abnormal electrograms.

Accordingly, procedural endpoints should extend beyond VT noninducibility and incorporate accurate identification of abnormal conduction regions exhibiting wavefront discontinuity that are linked to the critical isthmus of the 3D VT circuit, followed by high-intensity ablation of these targeted sites. Ablation confined strictly to a single focal site is likely insufficient. Instead, ablation should encompass the critical site together with adjacent sites with wavefront discontinuities and local abnormal ventricular potentials within a defined surrounding area (Fig. 9).

However, the extent to which the ablation area should be expanded beyond the immediately targeted region, including adjacent zones where abnormal electrograms are recorded, remains uncertain. If the critical isthmus represents only the surface expression of a deeper 3D pathway, limiting ablation to a visually apparent focal site may fail to achieve durable circuit disruption. Given that the critical isthmus of a 3D VT circuit may not always be directly represented on the mapped surface, delivering ablation over an adequately sized area surrounding the targeted site may increase the likelihood of intersecting a more superficially traversing component of the intramural isthmus. It should be acknowledged, however, that current data are insufficient to establish an optimal upper limit for the extent of the ablation area. Future prospective studies will be required to determine the minimal yet sufficient ablation area necessary to effectively suppress VT recurrence, although this may vary depending on the underlying cardiac pathology, intraprocedural hemodynamic stability, and VT inducibility. However, it appears prudent to avoid indiscriminate extension of ablation into normal-voltage myocardium lacking abnormal electrograms, even when the targeted site is situated within a scar border zone adjacent to preserved myocardium.

### 7.3 The Importance of Post-Ablation Remapping

If the objective of ablation is disruption of a 3D reentrant substrate, post-ablation assessment should confirm modification of the underlying conduction architecture rather than merely the disappearance of isolated electrogram abnormalities.

Regardless of whether a broad modification strategy such as scar homogenization or core isolation is employed, or a more selective targeted ablation approach focusing on DZs or RAP is applied, post-ablation remapping plays a critical role in procedural assessment. In the context of targeted ablation, the key objective of remapping should not be limited to confirming the disappearance of abnormal electrograms. Rather, procedural endpoints should be evaluated from the perspective of whether the boundary conditions required to sustain a 3D reentrant circuit have been effectively disrupted.

Post-ablation remapping has been reported to demonstrate disappearance of RAP around the edge of LOB in approximately 70% of cases following ablation of targeted regions [38]. As RAP is generally localized adjacent to regions of latest activation, its disappearance may indicate interruption of downstream conduction pathways, in line with the concept of scar dechanneling. In addition, prior studies have demonstrated that endocardial ablation can, in selected cases, achieve transmural lesion formation, particularly in regions of reduced wall thickness, thereby eliminating epicardial substrate from the endocardial surface [78]. Furthermore, even in the absence of complete transmurality, endocardial ablation may disrupt interconnecting channels between endocardial and epicardial substrates, contributing to modification of intramural and subepicardial arrhythmogenic pathways.

These findings suggest that remapping can provide mechanistic confirmation of successful substrate modification. However, despite its intuitive appeal, robust evidence demonstrating that systematic post-ablation remapping with strategy modification based on remapping findings leads to improved long-term outcomes remains limited. Thus, post-ablation remapping should be viewed not simply as a confirmatory step, but as an integral component of a strategy aimed at validating 3D circuit interruption. Accordingly, further investigation is warranted to establish the prognostic value of remapping-guided procedural refinement.

### 7.4 Ablation of Intramural VT Circuits

In substrate-based ablation, the goal extends beyond acute termination of VT. Emphasis should instead be placed on analyzing activation patterns and identifying critical conduction abnormalities linked to the targeted VT. In contrast, when substrate mapping fails to reveal clear wavefront discontinuity, determination of an appropriate ablation endpoint becomes substantially more challenging.

**Fig. 10.**
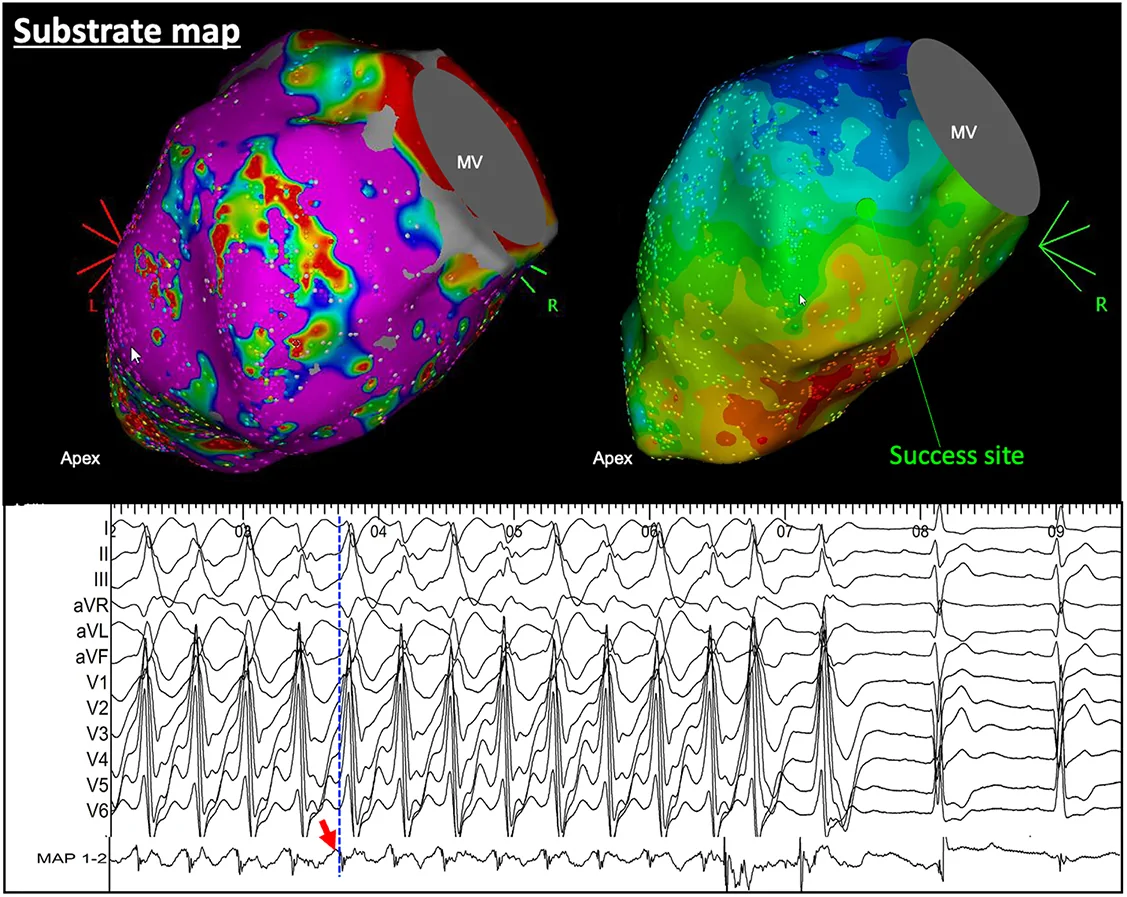
**VT ablation in the absence of overt activation slowing**. Substrate mapping demonstrated only a patchy low-voltage area without evidence of activation slowing. During VT, at the basal lateral left ventricle (green tag, upper panel), the near-field potential coincided with QRS onset (blue dotted line, lower panel), whereas a dull presystolic far-field potential was recorded (red arrow, lower panel). Radiofrequency ablation at this site resulted in immediate termination of the VT. Additional ablation encompassing the surrounding area was subsequently performed, after which the VT became noninducible. MV, mitral valve.

In the setting of predominantly intramural circuits, the disconnect between surface mapping findings and the true 3D isthmus becomes most apparent. Given the current lack of techniques that allow real-time and direct assessment of intramural conduction abnormalities, objective evaluation of ablation efficacy in such cases is limited. Under these circumstances, termination of VT during ablation and subsequent noninducibility may represent the only reliable indicators of procedural success (Fig. 10).

Several alternative ablation strategies aimed at achieving greater lesion depth have been explored, including bipolar ablation, chemical ablation, high-power radiofrequency delivery using open-irrigated electrodes cooled with half-normal saline, and ultra–low-temperature cryoablation [79,80,81,82,83,84]. Among emerging energy modalities, pulsed field ablation (PFA) has attracted considerable interest owing to its myocardial selectivity and potential for deep lesion formation. By inducing irreversible electroporation, PFA creates lesions through a nonthermal mechanism, raising the possibility of modifying 3D arrhythmogenic substrates while minimizing collateral injury to adjacent structures such as coronary arteries, neural tissue, and the esophagus. Consistent with this rationale, accumulating evidence has begun to suggest the potential efficacy of PFA in the treatment of VT [85,86,87,88,89]. Future studies will be essential to clarify the role of PFA and other deep-penetrating ablation modalities in addressing intramural VT circuits and in redefining procedural endpoints for scar-related VT ablation.

However, irrespective of the technique or energy modality used, in the absence of established methods for direct intraprocedural assessment of intramural conduction abnormalities, VT termination during ablation and subsequent noninducibility may continue to serve as the most dependable indicators of procedural success.

## 8. Future Perspectives

As the conceptual framework of scar-related VT shifts from a predominantly planar interpretation toward a volumetric 3D paradigm, future advances must aim not only at improving lesion delivery but also at enhancing visualization of the underlying conduction architecture.

Further refinement of our understanding of 3D arrhythmogenic substrates will require closer integration of preprocedural cardiac imaging with 3D electroanatomical mapping. Beyond purely structural assessment of scar, emerging imaging approaches have begun to incorporate functional perspectives, such as evaluation of regional activation slowing and conduction heterogeneity using advanced cardiac imaging techniques [90,91,92]. These functional imaging modalities may further enhance identification of arrhythmogenic substrates by bridging the gap between structural abnormalities and electrophysiological behavior.

Looking ahead, the integration of artificial intelligence and machine learning with cardiac imaging and high-resolution 3D electroanatomical mapping holds substantial promise. Through computational modeling and simulation, these approaches may enable more sophisticated characterization of arrhythmia initiation and maintenance mechanisms, ultimately facilitating personalized ablation strategies tailored to patient-specific 3D substrates [93,94,95,96,97,98]. Ultimately, convergence of advanced imaging, mechanistically informed targeting, and precise energy delivery may redefine both the strategy and the procedural endpoints of scar-related VT ablation.

## 9. Limitations

Targeted ablation strategies based on functional substrate mapping have several limitations. First, as noted above, this approach may be less applicable in cases of predominantly intramural (mid-myocardial) arrhythmogenic substrate, in which no obvious wavefront discontinuity related to the targeted VT can be identified within the mapped chamber. Second, functional substrate mapping requires high-density and high-resolution mapping; therefore, its accuracy is influenced not only by the mapping system and catheter but also, to a certain extent, by the skill and experience of the operator and mapper. Third, further investigation is needed to compare functional substrate–guided targeted ablation with conventional structural substrate–based ablation in terms of efficacy and safety, as well as to determine which patient populations may derive the greatest benefit from targeted approaches.

## 10. Conclusions

This review highlights that, within a three-dimensional perspective of scar-related VT substrate, accurate identification of critical conduction abnormalities as ablation targets is essential. In this context, procedural assessment should be reconsidered to focus on effective disruption of these abnormalities, supported by post-ablation remapping, as a reflection of successful modification of the underlying three-dimensional substrate, rather than relying solely on conventional endpoints such as VT noninducibility. Intramural substrates remain a major challenge, underscoring the need for improved methods to assess intramural conduction and to achieve durable lesion formation.
